# Instance Transfer Learning with Multisource Dynamic TrAdaBoost

**DOI:** 10.1155/2014/282747

**Published:** 2014-07-24

**Authors:** Qian Zhang, Haigang Li, Yong Zhang, Ming Li

**Affiliations:** School of Information and Electrical Engineering, China University of Mining and Technology, Xuzhou, Jiangsu 221116, China

## Abstract

Since the transfer learning can employ knowledge in relative domains to help the learning tasks in current target domain, compared with the traditional learning it shows the advantages of reducing the learning cost and improving the learning efficiency. Focused on the situation that sample data from the transfer source domain and the target domain have similar distribution, an instance transfer learning method based on multisource dynamic TrAdaBoost is proposed in this paper. In this method, knowledge from multiple source domains is used well to avoid negative transfer; furthermore, the information that is conducive to target task learning is obtained to train candidate classifiers. The theoretical analysis suggests that the proposed algorithm improves the capability that weight entropy drifts from source to target instances by means of adding the dynamic factor, and the classification effectiveness is better than single source transfer. Finally, experimental results show that the proposed algorithm has higher classification accuracy.

## 1. Introduction

In data mining, a general assumption for the traditional machine learning is that training data and test data have the same distribution. However, in the practical application, this assumption cannot be often met [1]. By transferring and sharing different field knowledge for target task learning, transfer learning makes the traditional learning from scratch an addable one. This must improve the learning efficiency and reduce the learning cost [2, 3]. In 2005, Information Processing Techniques Office (IPTO) gave a new mission of transfer learning: the ability of a system to recognize and apply knowledge and skills learned in previous tasks to novel tasks. In this definition, transfer learning aims to extract the knowledge from one or more source tasks and apply the knowledge to a target task [2]. Since the transfer learning needs to use information from similar domains and tasks, its effectiveness is related to the correlation between the source and target domains.

However, transfer learning is more complex than traditional machine learning because of the introduction of transfer. There are many kinds of knowledge representation in related domains, such as sample instances, feature mapping, model parameters, and association rules. Due to the simpleness of implement, the paper selects sample instances as knowledge representation to design the effective transfer algorithm. In detail, instance transfer learning is used to improve the classification accuracy by finding training samples in other source domains which have strong correlation with the target domain and reusing them in the learning of target task [4]. Obviously, how to decide weight of this training data should influence the effectiveness of candidate classifiers [5].

Up to now, researchers have proposed several approaches to solve transfer learning problems. Ben and Schuller provided a theoretical justification for multitask learning [6]. Daumé and Marcu studied the domain-transfer problem in statistical natural language processing by using a specific Gaussian model [7]. Wu and Dietterich proposed an image classification algorithm by using both inadequate training data and plenty of low quality auxiliary data [8]. This algorithm demonstrates some improvement by using the auxiliary data, but it does not give a quantitative study using different auxiliary examples. Liao et al. proposed a new active learning method to select the unlabeled data in a target domain to be labeled with the help of the source domain data [9]. Rosenstein et al. proposed a hierarchical Naive Bayes approach for transfer learning by using auxiliary data and discussed the applying time problem of transfer learning [10].

Transfer AdaBoost algorithm, also called TrAdaBoost, is a classic transfer learning algorithm which is proposed by Dai et al. [11]. TrAdaBoost assumes that the source and target domain data use exactly the same set of features and labels, but the distributions of the data in the two domains are different. In addition, TrAdaBoost assumes that, due to the difference in distributions between the source and the target domains, some of the source domain data may be useful in learning for the target domain but some of them may not and could even be harmful. Since TrAdaBoost relies only on one source, its learning effects will become poor when there is a weak correlation between the source and target domains. Moreover, as the literatures [12–14] said, TrAdaBoost has the weaknesses of weight mismatch, introducing imbalance and rapid convergence of source weights. The purpose of this paper is to remove the weight drift phenomenon efficiently, improve learning efficiency, and inhibit the negative transfer.

## 2. Multisource Dynamic TrAdaBoost Algorithm

Considering the correlation between multiple source domains and the target domain, recently Yao and Doretto proposed multisource TrAdaBoost (MSTrA) transfer learning algorithms [15]. As an instance-based transfer learning method, MSTrA selects its training samples from different source domains. At each iteration, MSTrA always selects the most related source domain to train the weak classifier. Although this can ensure that the knowledge transferred is relevant to the target task, MSTrA ignores effects of other source domains. Samir and Chandan proposed an algorithm (DTrAdaBoost) with an integrated dynamic cost to resolve a major issue in the boosting-based transfer algorithm, TrAdaBoost [16]. This issue causes source instances to converge before they can be used for transfer learning. But DTrAdaBoost has low efficiency of learning.

In order to overcome the above disadvantage, a multisource dynamic TrAdaBoost algorithm (MSDTrA) is proposed. By using this algorithm, the rate of convergence of source sample weight will be reduced based on weak correlation to target domain [17]. Supposing there are *N* source domains, *D*_*S*_1__,…, *D*_*S*_*N*__; *N* source tasks, *T*_*a*_1__,…, *T*_*a*_*N*__; and *N* source training data, *D*_*a*_1__,…, *D*_*a*_*N*__, the purpose of transfer learning is to make good use of them to improve the learning effectiveness of the target classifier function ${\hat{f}}_{b}:X\to Y$. In detail, the algorithm steps of MSDTrA are described as follows.

**Step 1 .**

Initialize the weight vector (*ω*_*a*_1__,…, *ω*_*a*_*N*__, *ω*_*b*_), where *ω*_*a*_*k*__ = (*ω*_*a*_*k*__^1^,…, *ω*_*a*_*k*__^*n*_*a*_*k*__^) are the weight vectors of training samples with *k*th source domain and *ω*_*b*_ = (*ω*_*b*_^1^,…, *ω*_*b*_^*n*_*b*_^) are the weight vectors of training samples in target domain.

**Step 2 .**

Set the value of *β*_*a*_ as follows:

(1) $$\begin{array}{c} \beta_{a}=\frac{1}{1+\sqrt{2*\ln\left(n_{a}\right)/M}}, \end{array}$$

where *n*_*a*_ = ∑_*k*_ *n*_*a*_*k*__ is the number of all source domains training samples and *n*_*a*_*k*__is the sample number of training sets with *k*th source domain.

**Step 3 .**

Empty the set of candidate weak classifiers and normalize the weight vectors (*ω*_*a*_1__,…, *ω*_*a*_*N*__, *ω*_*b*_) to 1.

**Step 4 .**

Select a base learner to obtain the candidate weak classifiers (*f*_*b*_^*t*^)^*k*^ based on training set *D*_*a*_*k*__ ∪ *D*_*b*_; calculate the error of (*f*_*b*_^*t*^)^*k*^ on *D*_*b*_ according to the following equation:

(2) $$\begin{array}{c} {\left(\varepsilon_{b}^{t}\right)}^{k}=\overset{n_{b}}{\underset{j=1}{\sum }}\frac{\omega_{b}^{j}\sum_{k=1}^{N}\left[y_{b}^{j}\neq {\left(f_{b}^{t}\right)}^{k}\cdot x_{b}^{j}\right]}{\sum_{i=1}^{n_{b}}\omega_{b}^{i}}; \end{array}$$

update the weight of (*f*_*b*_^*t*^)^*k*^ by using the vectors update strategy:

(3) $$\begin{array}{c} {\left(\omega_{b}^{t}\right)}^{k}=\frac{e^{1-{\left(\varepsilon_{b}^{t}\right)}^{k}}}{e^{{\left(\varepsilon_{b}^{t}\right)}^{k}}}. \end{array}$$

Repeat the above method until all source domains are traversed, where (*ε*_*b*_^*t*^)^*k*^ is the error rate of candidate weak classifiers with *k*th source domains in target domain. *y*_*b*_^*j*^ ≠ (*f*_*b*_^*t*^)^*k*^ · *x*_*b*_^*j*^ stands for error classified with the candidate weak classifiers. According to the vectors update strategy above, the error of each weak classifier in the target training set is computed and a weight is assigned to each weak classifier according to the error. The larger the error is, the smaller the weight becomes. In other words, source domains which correspond to those classifiers with high classification accuracy contain much valuable information for the learning of target task.

**Step 5 .**

Integrate all weighted weak classifiers to obtain a candidate classifier at the *t*th iteration:

(4) $$\begin{array}{c} f_{b}^{t}=\underset{k}{\sum }\frac{{\left(\omega_{b}^{t}\right)}^{k}}{\sum_{k}{\left(\omega_{b}^{t}\right)}^{k}}{\left(f_{b}^{t}\right)}^{k}, \end{array}$$

where the classification error of *f*_*b*_^*t*^ on *D*_*b*_ at iteration *t* is

(5) $$\begin{array}{c} \varepsilon_{b}^{t}=\overset{n_{b}}{\underset{j=1}{\sum }}\frac{\omega_{b}^{j}\sum_{t=1}^{M}\left[y_{b}^{j}\neq f_{b}^{t}\cdot x_{b}^{j}\right]}{\sum_{i=1}^{n_{b}}\omega_{b}^{i}}, \end{array}$$

where *ε*_*b*_^*t*^ must be less than 0.5. Then, calculate the errors of the candidate classifier on the source and target training sets, based on which update the weights of training samples on the source and target domains. For the correct classified source training samples, their corresponding weights keep unchanged.

**Step 6 .**

Set

(6) $$\begin{array}{c} \beta_{b}^{t}=\frac{\varepsilon_{b}^{t}}{1-\varepsilon_{b}^{t}},\;C_{t}=2\left(1-\varepsilon_{b}^{t}\right),\;0\leq \varepsilon_{b}^{t}\leq \frac{1}{2}, \end{array}$$

where *C*_*t*_ = 2(1 − *ε*_*b*_^*t*^) is the expression of dynamic factor *C*_*t*_. And Theorem 1 will provide the deduce process.

**Step 7 .**

Update the weight vector of source samples according to the following rule:

(7) $$\begin{array}{c} \omega_{a_{k}}^{(t+1)\cdot i}=C_{t}\cdot \omega_{a_{k}}^{t\cdot i}\cdot {\left(\beta_{a}\right)}^{\sum_{t=1}^{M}\left[y_{b}^{j}\neq f_{b}^{t}\cdot x_{b}^{j}\right]},\;i\in D_{a_{k}}. \end{array}$$

Update the weight of target samples according to the rule:

(8) $$\begin{array}{c} \omega_{b}^{(t+1)\cdot i}=\omega_{b}^{t\cdot i}\cdot {\left(\beta_{b}^{t}\right)}^{\sum_{t=1}^{M}\left[y_{b}^{j}\neq f_{b}^{t}\cdot x_{b}^{j}\right]},\;i\in D_{b}, \end{array}$$

where the weight update of the source instances uses the weighted majority algorithm (WMA) mechanism. This updated mechanism is computed by *β*_*a*_ and *C*_*t*_. The target instance weights are updated by using *ε*_*b*_^*t*^, which is calculated on Step 6.

**Step 8 .**

Retrain all weak classifiers using the training samples with updated weights. If the maximum number of iterations is reached, *t* < *M*, return to Step 3; otherwise, turn to Step 9.

**Step 9 .**

Decide the final strong classifier

(9) $$\begin{array}{c} {\hat{f}}_{b}=\mathrm{sign}\left\{\overset{M}{\underset{t=1}{\prod }}\left[{\left(\beta_{b}^{t}\right)}^{-f_{b}^{t}}\right]-\overset{M}{\underset{t=1}{\prod }}\left[{\left(\beta_{b}^{t}\right)}^{-1/2}\right]\right\}. \end{array}$$

In the MSDTrA algorithm, TrAdaBoost's ensemble learning is selected to train classifiers based on the combination set of source and target instances in every step. WMA is used to adjust weights of the source set by decreasing the weight of misclassified source instances and preserving current weights of correctly classified source instances.

It can be seen from the above algorithm that the MSDTrA allows all source training samples to participate in learning process at each iteration, and different source training samples are assigned different weights. If a source training sample can improve the learning of target task, it will be assigned a large weight. Overall, the MSDTrA takes full advantage of all useful knowledge from all source domains, and this can obviously enhance the learning effectiveness of target task.

## 3. Theoretical Analysis

The previous section introduced in detail the proposed new algorithm, that is, the instance transfer learning algorithm. In this section, related theory analyses will be given according to single source TrAdaBoost algorithm [13]. First, Theorems 1 and 2 will proof the influence of source and target sample weight vectors with dynamic factor in source weight, respectively.

**Theorem 1 .**

A dynamic factor of *C*_*t*_ = 2(1 − *ε*_*b*_^*t*^) that is applied to the source weights can prevent their weight drift and get the weight vector to update mechanism of source sample.

**Proof**

Set *A* is sum of correctly classified target weights at boosting iteration *t* + 1 and *B* is sum of misclassified target weights at boosting iteration *t* + 1. Consider

(10) Anb·ωbt(1−εbt)·(εbt1−εbt)∑t=1M[ybj=fbt·xbj]=nb·ωbt(1−εbt)if  ∑t=1M[ybj=fbt·xbj]=0,Bnb·ωbt·εbt·(εbt1−εbt)∑t=1M[ybj=fbt·xbj]=na·ωakt(1−εbt)if  ∑t=1M[ybj≠fbt·xbj]=1.

Substituting for *A* and *B* to simplify the source update of TrAdaBoost, we have

(11) $$\begin{array}{c} \omega_{a_{k}}^{t+1}=\frac{\omega_{a_{k}}^{t}}{n_{a}\cdot \omega_{a_{k}}^{t}+A+B}=\frac{\omega_{a_{k}}^{t}}{n_{a}\cdot \omega_{a_{k}}^{t}+2\cdot n_{b}\cdot \omega_{b}^{t}\left(1-\varepsilon_{b}^{t}\right)}. \end{array}$$

Introducing the correction factor into the WMA, because of *ω*_*a*_*k*__^*t*+1^ = *ω*_*a*_*k*__^*t*^, we have

(12) $$\begin{array}{c} \omega_{a_{k}}^{t}=\frac{C_{t}\cdot \omega_{a_{k}}^{t}}{C_{t}\cdot n_{a}\cdot \omega_{a_{k}}^{t}+2\cdot n_{b}\cdot \omega_{b}^{t}\left(1-\varepsilon_{b}^{t}\right)},\\ C_{t}=\frac{2\cdot n_{b}\cdot \omega_{b}^{t}\left(1-\varepsilon_{b}^{t}\right)}{1-n_{a}\cdot \omega_{a_{k}}^{t}}=\frac{2\cdot n_{b}\cdot \omega_{b}^{t}\left(1-\varepsilon_{b}^{t}\right)}{n_{b}\cdot \omega_{b}^{t}}=2\left(1-\varepsilon_{b}^{t}\right). \end{array}$$

**Theorem 2 .**

The dynamic factor of *C*_*t*_ = 2(1 − *ε*_*b*_^*t*^) that is applied to the source weights makes the target weights converge as outlined by TrAdaBoost.

**Proof**

In TrAdaBoost, without any source instances (*n*_*a*_ = 0), target weights for correctly classified instances will be updated as

(13) ωbt+1=ωbt∑j=1nbωbt·(εbt/(1−εbt))∑t=1M[ybj≠fbt·xbj]=ωbtA+B=ωbt2·nb·ωbt(1−εbt)=ωbt2(1)(1−εbt).

Applying the dynamic factor to update the source instance weight, we can get the update mechanism of the target instance weight based on MSDTrA. Consider

(14) ωbt+1=ωbtna·ωakt+2nb·ωbt(1−εbt)=ωbtCt·na·ωakt+2nb·ωbt(1−εbt)=ωbt2(1−εbt)·na·ωakt+2nb·ωbt(1−εbt)=ωbt2(1−εbt)(na·ωakt+nb·ωbt)=ωbt2(1−εbt)(1).

Next, we analysis the performance of MSDTrA on the target training set.

**Theorem 3 .**

The final error on the target training set is

(15) $$\begin{array}{c} \varepsilon \leq 2^{M}\cdot \overset{M}{\underset{t=1}{\prod }}\sqrt{\varepsilon_{b}^{t}\left(1-\varepsilon_{b}^{t}\right)}. \end{array}$$

**Proof**

Supposing that the final sample set which contains all misclassified samples on the target domain is *T*, the final error is *ε* = |*T*|/*n*_*b*_.

At each iteration, the error on the target training set is

(16) $$\begin{array}{c} \varepsilon_{b}^{t}=\underset{k}{\sum }\frac{{\left(w_{b}^{t}\right)}^{k}}{\sum_{k}{\left(w_{b}^{t}\right)}^{k}}{\left(\varepsilon_{b}^{t}\right)}^{k}=\frac{\sum_{k}e^{1-2{\left(\varepsilon_{b}^{t}\right)}^{k}}{\left(\varepsilon_{b}^{t}\right)}^{k}}{\sum_{k}e^{1-2{\left(\varepsilon_{b}^{t}\right)}^{k}}}, \end{array}$$

where 0 ≤ (*ε*_*b*_^*t*^)^*k*^ ≤ 1/2.

If the error on the target training set is 0, *ε*_*b*_^*t*^ = 0, training sample weights are not updated, *w*_*b*_^(*t*+1)*i*^ = *w*_*b*_^*ti*^. If *ε*_*b*_^*t*^ ≠ 0 and *β*_*b*_^*t*^ = *ε*_*b*_^*t*^/(1 − *ε*_*b*_^*t*^) ≠ 0, the updating rule for the weights of target training samples is as follows:

(17) ∑i=1nbwb(t+1)i=∑i=1nbwbt·i(βbt)1−εbt≤∑i=1nbwbt·i(1−(1−εbt)βbt).

Then,

(18) ∑i=1nbwb(M+1)i=∑i=1nbwbt·i(βbt)1−εbt≤∑i=1nbwbi∏t=1M(1−(1−εbt)βbt)≔∏t=1M(1−(1−εbt)βbt).

In addition, we have the following criterion:

(19) $$\begin{array}{c} \overset{n_{b}}{\underset{i=1}{\sum }}w_{b}^{(M+1)i}\geq \underset{i\in S}{\sum }w_{b}^{i}{\left(\overset{M}{\underset{t=1}{\prod }}\beta_{b}^{t}\right)}^{1/2}=\varepsilon {\left(\overset{M}{\underset{t=1}{\prod }}\beta_{b}^{t}\right)}^{1/2}. \end{array}$$

Combining (18) and (19), we have

(20) $$\begin{array}{c} \varepsilon {\left(\overset{M}{\underset{t=1}{\prod }}\beta_{b}^{t}\right)}^{1/2}\leq \overset{M}{\underset{t=1}{\prod }}\left(1-\left(1-\varepsilon_{b}^{t}\right)\beta_{b}^{t}\right). \end{array}$$

Substituting *β*_*b*_^*t*^ = *ε*_*b*_^*t*^/(1 − *ε*_*b*_^*t*^) into (20), we can obtain

(21) $$\begin{array}{c} \varepsilon \leq 2^{M}\cdot \overset{M}{\underset{t=1}{\prod }}\sqrt{\varepsilon_{b}^{t}\left(1-\varepsilon_{b}^{t}\right)}. \end{array}$$

According to Theorem 3, because the condition of *ε*_*b*_^*t*^ < 0.5 is satisfied in the algorithm, the error in final target training data will decrease with the increase of iterations. And the upper bound of the associated generalization error can be calculated by $\varepsilon +O(\sqrt{{Md}_{\text{VC}}/n_{b}})$, where *d*_VC_ is the VC-dimension of the weak classifier model.

## 4. Experimental Results and Analysis

The performance of the proposed method is investigated based on object category recognition in this section. Without loss of generality, we consider the following case: a small number of training samples of a target object category and a large number of training samples of other source object categories. For any test sample, we verify whether it belongs to the target object category or not.

### 4.1. Experimental Setting

For object category recognition, the Caltech 256 datasets that contain 256 object categories are considered. Practically, among 256 object categories, the 80 categories that contain more than 50 samples are used in our experiment. We designate the target category and randomly draw the samples that form the target data. The number of samples for training *n*_*b*_ is limited between 1 and 50, while the number of samples for testing is 50. Furthermore, in order to illustrate the proposed method does not depend on the data set, we have also used the background dataset, collected via the Google image search engine, along with the remaining categories as our augmented background data set, to verify the effectiveness and robustness of this method.

The remaining categories are treated as the repository from which to draw positive samples for the source data. The numbers of source categories or domains are varied from 1 to 10 in order to investigate the performance of the classifiers with respect to the variability of domains. The number of samples for one source of data is 100. For each target object category, the performance of the classifier is evaluated over 20 random combinations of *N* source object categories. Given the target and source categories, the performance of the classifier is obtained by averaging over 20 trials of experiments. The overall performance of the classifier is averaged over 20 target categories. SVM is selected as base classifiers and the iteration is 50.

### 4.2. Error Analysis

**Figure 1:**
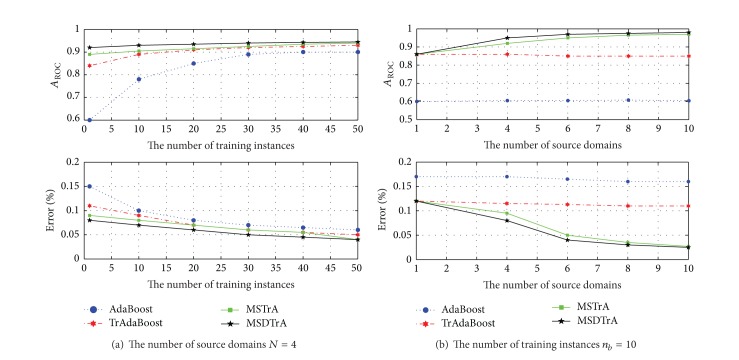
Performance comparison.

Since transfer learning is not needed to get good classification results when the target data set is large, standard cross-validation method is not used here. Small portion data of the target set are used for training, and most of the remaining samples are used for testing.  Figure 1 compares AdaBoost, TrAdaBoost, MSTrA, and MSDTrA based on the area under the receiver operating characteristic curve (ROC) with different number of target training samples (*n*_*b*_ = {1,10,20,30,40,50}) and different number of sources in the field (*N* = {1,4, 6,8, 10}). Moreover, for the area bounded by the ROC curve and the *X*-axis, *A*_ROC_ is used to evaluate the performance of different algorithms.

Practically, fixing the number of source domains *N* = 4, Figure 1(a) shows the ROC curves of the four algorithms with the increase of the number of training instances. Since AdaBoost does not transfer any knowledge from the source, its performance depends mainly on the number of *n*_*b*_. For a very small value of *n*_*b*_, it performs slightly improvement as the ROC curves show. However, due to the transfer learning mechanism, TrAdaBoost has good improvement by combining the three sources. By incorporating the ability to transfer knowledge from multiple individual domains, MSTrA and MSDTrA demonstrate a significant improvement in recognition accuracy, even for a very small *n*_*b*_. In addition, the performance of AdaBoost and TrAdaBoost strongly depends on the selection of source domains and target positive samples, as the standard deviation of *A*_ROC_ shows.

Fixing the number of training instances *n*_*b*_ = 10, Figure 1(b) shows the ROC curves of the four algorithms with increase of the number of source domains. We can see that as the number of source domains increase, the *A*_ROC_ of MSTrA and MSDTrA increases and the corresponding standard deviations also decrease. This indicates an improved performance in both accuracy and consistency. Since TrAdaBoost is incapable of exploring the decision boundaries separating multiple source domains, its performance keeps unchanged regardless of the number of source domains.

**Figure 2:**
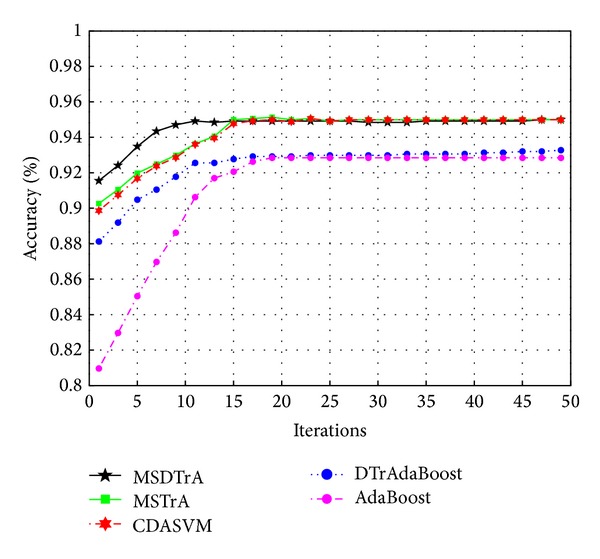
The classification performance on the target domain.

Figure 2 compares the classification performance of different methods in the target domain. We can see that AdaBoost algorithm does not transfer source domain knowledge and gets lower classification accuracy. DTrAdaBoost has relatively poor test results, because it only uses one source domain training sample set and gains the least useful knowledge from source domain. CDASVM based on structural risk minimization model fully considers the source domain sample information, and thus it has good classification accuracy. MSTrA and MSDTrA use four different sources domain training sets which contain more useful information, so they get higher testing accuracy than other algorithms. In each set of experiments, MSTrA only selects classification with the highest accuracy at each iteration and ignores the impact of other source domains to target tasks. But by adding the dynamic factors and weighting mechanism, MSDTrA makes better use of all sources domains useful knowledge and eliminates the influences of unrelated samples in sources domains training set to target tasks, so it has better performance than MSTrA algorithm.

In order to have objective and scientific comparison results, hypothesis testing is used on the experimental results. Let the variables *X*_1_, *X*_2_, *X*_3_, *X*_4_, *X*_5_ denote the classification error rate of MSDTrA, MSTrA, CDASVM, DTrAdaBoost, and AdaBoost algorithms, respectively. Since the value of *X*_1_, *X*_2_, *X*_3_, *X*_4_, *X*_5_ is subject to many random factors, we assume that they submit to normal distribution, *X*_*i*_ ~ *N*(*μ*_*i*_, *σ*_*i*_^2^), *i* = 1,2, 3,4, 5. Now, we compare the random variable means of these algorithms, *μ*_*i*_ (*i* = 1,2, 3,4, 5). The smaller the *μ*_*i*_ is, the lower the expected classification error rate is and the higher the efficiency is. Because the sample variance is the unbiased estimation of the overall variance, the sample variance value is used as an estimate of the generality variance. In this experiment the significance level *α* is set as 0.01.

Table 1 shows the comparison process on *μ*_*i*_ and other parameters. We can see from Table 1 that the expectations of classification error rate in MSDTrA is far below than other algorithms.

### 4.3. Time Complexity

**Figure 3:**
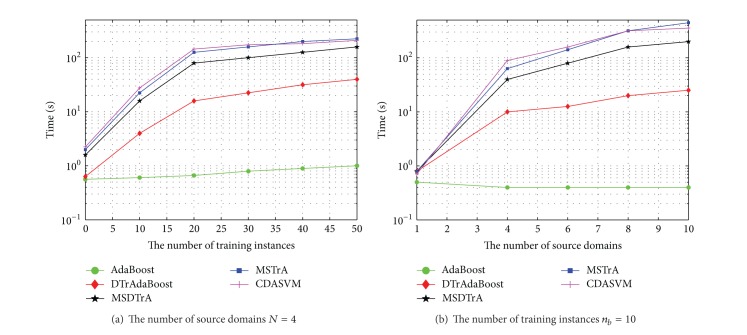
Time cost comparison on different methods.

Since several domains are used into the learning of target task together, time complexity of multisource domains is more than single domain. Supposing that the time complexities of training a classifier and updating weight are *C*_*h*_ and *C*_*w*_, respectively, the time complexity of AdaBoos, DTrAdaBoost, MSTrA, and MSDTrA can be approximated to *C*_*h*_ *O*(*M*) + *C*_*w*_ *O*(*n*_*b*_ *M*),  *C*_*h*_ *O*(*M*) + *C*_*w*_ *O*(*n*_*a*_ *M*),  *C*_*h*_ *O*(*NM*) + *C*_*w*_ *O*(*n*_*a*_ *NM*) and *C*_*h*_ *O*(*NM*) + *C*_*w*_ *O*(*n*_*a*_ *M*). Furthermore, Figure 3 shows the average training time of the four algorithms with fixed *N*, *n*_*b*_.

### 4.4. Dynamic Factor

**Figure 4:**
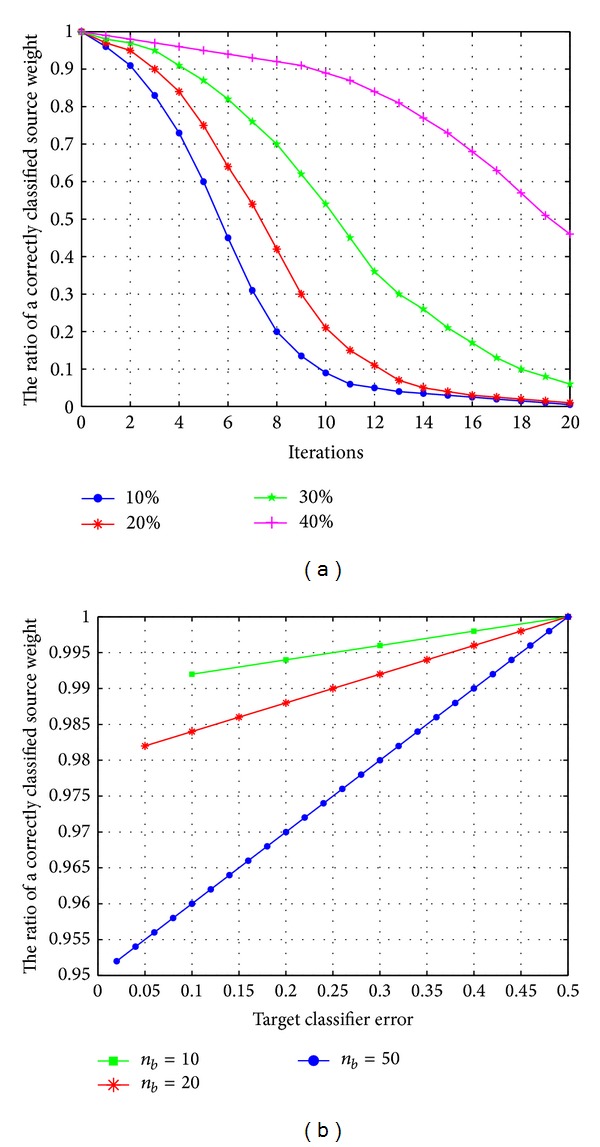
(a) Results for 20 iterations with different target error rates. (b) Results after a single iteration with different number of target instances.

This experiment will prove the effect of dynamic factor on source weights and target weights. Here a sources domain is considered, *N* = 1. In Figure 4(a), the number of instances is set as constant (*n*_*a*_ = 1000, *n*_*b*_ = 200) and the source error rate is set to zero. According to the WMA, the weights should not change because of *ε*_*a*_*k*__^*t*^ = 0; that is, *ω*_*a*_*k*__^*t*+1^ = *ω*_*a*_*k*__^*t*^. When target error rates *ε*_*b*_^*t*^ = {10%, 20%, 30%, 40%}, the ratio of the weights of MSDTrA and MSTrA is plotted at different boosting iterations.

We can see from Figure 4(a) the following. (1) In MSTrA, source weights converge always even the classification results are correct. (2) MSDTrA matches the behavior of the WMA. (3) If dynamic factor is not applied, the smaller the value of *ε*_*b*_^*t*^ is and the faster the convergence rate of source weights is. In addition, for a weak learner with *ε*_*b*_^*t*^ = 10%, MSTrA is still not able to get good performance by using over 1000 source instances, even though they were never misclassified.

### 4.5. Rate of Convergence

The number of source instances was set (*n*_*b*_ = 1000), and the classification error is permitted to vary within the range of *ε*_*b*_^*t*^ ∈ {10% ~ 50%}; Figure 4(b) shows results after a single iteration with different number of target instances {10,20,50}. It can be observed that after a single boosting iteration, the ratio of a correctly classified source instances increases with the increases of *ε*_*b*_^*t*^.

## 5. Conclusions

Considering the situation that sample data from the transfer source domain and the target domain have similar distribution, an instance transfer learning method based on multisource dynamic TrAdaBoost is provided. By integrating with the knowledge in multiple source domains, this method makes good use of the information of all source domains to guide the target task learning. Whenever candidate classifiers are trained, all the samples in all source domains are involved in learning, and the information that is beneficial to target task learning can be obtained, so that negative transfer can be avoided. The theoretical analysis and experimental results suggest that the proposed algorithm has higher classification accuracy compared with several existing algorithms.

## Figures and Tables

**Table 1:** Hypothesis testing for experimental results.

Hypothesis	$\begin{array}{c} H_{0}\text{:}{\;\mu }_{1}\geq \mu_{2}\\ H_{1}\text{:}\;\mu_{1}<\mu_{2} \end{array}$	$\begin{array}{c} H_{0}\text{:}\;\mu_{1}\geq \mu_{3}\\ H_{1}\text{:}\;\mu_{1}<\mu_{3} \end{array}$	$\begin{array}{c} H_{0}\text{:}\;\mu_{1}\geq \mu_{4}\\ H_{1}\text{:}\;\mu_{1}<\mu_{4} \end{array}$	$\begin{array}{c} H_{0}\text{:}{\;\mu }_{1}\geq \mu_{5}\\ H_{1}\text{:}{\;\mu }_{1}<\mu_{5} \end{array}$

Statistics	$U_{1}=\frac{{\overset{-}{X}}_{1}-{\overset{-}{X}}_{2}}{\sqrt{\sigma_{1}^{2}/n_{1}+\sigma_{2}^{2}/n_{2}}}$	$U_{2}=\frac{{\overset{-}{X}}_{1}-{\overset{-}{X}}_{3}}{\sqrt{\sigma_{1}^{2}/n_{1}+\sigma_{3}^{2}/n_{3}}}$	$U_{3}=\frac{{\overset{-}{X}}_{1}-{\overset{-}{X}}_{4}}{\sqrt{\sigma_{1}^{2}/n_{1}+\sigma_{4}^{2}/n_{4}}}$	$U_{4}=\frac{{\overset{-}{X}}_{1}-{\overset{-}{X}}_{5}}{\sqrt{\sigma_{1}^{2}/n_{1}+\sigma_{5}^{2}/n_{5}}}$

Rejection region	*U*_1_ ≤ −*Z*_*α*_ = −2.325	*U*_2_ ≤ −*Z*_*α*_ = −2.325	*U*_3_ ≤ −*Z*_*α*_ = −2.325	*U*_4_ ≤ −*Z*_*α*_ = −2.325

Value of the statistic	*U*_1_ = −58.67	*U*_2_ = −114.56	*U*_3_ = −136.59	*U*_4_ = −158.23

Conclusion	*H*_1_: *μ*_1_ < *μ*_2_	*H*_1_: *μ*_1_ < *μ*_3_	*H*_1_: *μ*_1_ < *μ*_4_	*H*_1_: *μ*_1_ < *μ*_5_
